# Multiple Ant Species Tending Lac Insect *Kerria yunnanensis* (Hemiptera: Kerriidae) Provide Asymmetric Protection against Parasitoids

**DOI:** 10.1371/journal.pone.0098975

**Published:** 2014-06-02

**Authors:** Youqing Chen, Zhixing Lu, Qiao Li, Benjamin D. Hoffmann, Wei Zhang

**Affiliations:** 1 Research Institute of Resources Insects, Chinese Academy of Forestry, Kunming, Yunnan, China; 2 School of Forestry, Southwest Forestry University, Kunming, Yunnan, China; 3 Tropical Ecosystems Research Centre, CSIRO Ecosystem Sciences, Darwin, NT, Australia; Stanford University, United States of America

## Abstract

This study investigated the effects of ant attendance on the parasitoid community and parasitism of lac insect *Kerria yunnanensis* aggregations in Yunnan province, China. We manipulated ant attendance to establish three treatments: (1) ant exclusion; (2) low ant attendance by several ant species; and (3) high ant attendance by *Crematogaster macaoensis*. Five parasitoid species were collected, with two species contributing 82.7 and 13.2% of total abundance respectively. Total parasitoid abundance was lowest in the February sample when *K. yunnanensis* was in its younger life stage, being significantly lower in the ant exclusion treatment. In April, all three treatments had significantly different parasitoid abundances, being highest in the ant exclusion treatment and the lowest in the high ant attendance treatment. When ants were present, there were strong negative relationships between total parasitoid abundance and ant abundance, with the relationships being dependent upon the ant species composition and abundance. The patterns of total parasitoid abundance were driven by the two most abundant parasitoid species. Parasitoid species richness did not differ among treatments or between sample times, however, multivariate analysis confirmed that overall parasitoid community structure differed significantly among treatments and between sample times, with the high ant attendance treatment differing most from the other two treatments. Interestingly the absence of ants did not result in increased parasitism from four of the five parasitoids. Ants in lac insect farming systems have a clear role for agricultural pest management. A full understanding of the asymmetric abilities of ants to influence parasitoid communities, and affect parasitism of hosts will require further experimental manipulation to assess the relative roles of 1) the abundance of each individual ant species on parasitoid access to hosts, 2) competition among parasitoids, and 3) the interaction between the first two factors.

## Introduction

Honeydew-producing insects and ants have many mutualistic associations, in which ants obtain honeydew that phytophagous insects exude [1], and in return, the ants protect the symbionts from their natural enemies [2], [3], [4], [5]. The strength of associations between ant and honeydew-producing insects, as well as the ability for ants to protect their symbionts from predators and parasites, vary greatly, even for the same pair of species among habitats [6]. Factors influencing these partnerships include the species of tending ants [7], [8], [9], [10], the aggregation size of the honeydew-producing insects [11], [12], temperature [13], the developmental stage of the honeydew-producing insects [12], [14], as well as competition among honeydew-producing insect aggregations for the services of ant mutualists [12], [15], [16], [17]. Understanding these dynamics is important to improve our management of ecosystems and agricultural systems alike.

Within agroecosystems, the protection of phytophagous insects by ants is generally considered to be detrimental to crop production, because phytophagous insects can attain high densities that reduce crop plant productivity [18]. Unique among agricultural practices is the farming of lac insects (*Kerria* spp., Hemiptera: Kerriidae) for lac, a red resin produced by the insects that protects them from water loss, waterlogging from rain, predation, and contamination with their own honeydew [1], [19]. The resin is utilized widely by food, textiles and pharmaceutical industries. In China, lac production is worth US$92 million annually, and the lac insect most commonly cultivated is *K. yunnanensis* [20].

Lac cultivation is unique among most agricultural practices in that it is obliged to be organic, because chemicals that can be used to kill predators and parasitoids of lac insects would also kill the lac insects. Many ant species tend *K. yunnanensis* for honeydew [21], and the presence of at least one species in China, *Crematogaster macaoensis*, was recently found to improve key features of fitness of *K. yunnanensis*, including survival rate, number of females and number of offspring [22], presumably by providing protection from predators and parasitoids. Therefore ants have a clear role in the management of lac cultivation.

Despite the economic importance of this agricultural system, and a clear reliance on ants for lac production, very little is known about lac insect-ant-parasitoid relationships. All other work conducted to date has been limited to the identification and basic biology of parasitoids [23], [24], [25], [26], [27], and demonstrating the impacts of parasitoids on lac insects [22], [28], [29]. Here, for the first time, we investigate the role of ants in protecting lac insects from parasitoids within the typical conditions of a lac farming system. Specifically we address two hypotheses: 1) that different ant species with different abundance levels provide different levels of protection to *K. yunnanensis*; and 2) that these relationships differ with the different life stages of *K. yunnanensis*.

## Methods

The study was carried out on private land; we confirmed that the owner of the land gave permission to conduct the study on this site.

### Study system

The research was conducted in a lac insect agroecosystem in Mojiang county, Yunnan Province, SW China (101°43′ E, 23°14′ N). The study region is characterized by an average annual rainfall of 1500–2100 mm (mostly in May to October) and a mean annual temperature of 18.2°C. The lac insect agroecosystem is the main form of cultivation in mountainous regions between altitudes of 900–1500 m. The experiments were performed in a 10 ha plantation dominated by *Dalbergia obtusifolia*, but also containing *Ficus semicordata* and *D. szemaoensis,* with a density of 1500 trees per hectare, and with corn grown between the trees. No permit was required to conduct sampling and the field studies did not involve endangered or protected species.

### Experimental design

At experimental commencement in October 2009, one third of the trees throughout the plantation were inoculated with *K. yunnanensis*, typical of normal lac farming practices. Inoculation involved placing brood lac, containing *K. yunnanensis* larvae, on branches. The larvae move from the brood lac onto the branches and aggregate.

We selected 174 *D. obtusifolia* that were eight years old, 2.5 m to 2.8 m high, with a trunk diameter of 5 cm to 7 cm, and spaced at least 20 m apart. For the ant exclusion treatment,prior to *K. yunnanensis* inoculation, all ants visiting or living on 60 trees were removed, and a ring barrier of insect glue was applied to the main branch 0.5 m above the ground to prevent ant access. All other vegetation touching the tree were also cleared. Every second week the insect glue was replaced and the surrounding vegetation was cleared to maintain ant exclusion.

Sixty trees were designated to the mixed ant species low ant attendance treatment (hereafter referred to as low ant attendance treatment) because they had multiple ant species, such as *Crematogaster macaoensis*, *Dolichoderus thoracicus*, *Camponotus parius*, and *Polyrhachis tibialis*, occasionally visiting *K. yunnanensis* for honeydew, typically as individuals. For the *C. macaoensis* high ant attendance treatment (hereafter referred to as the high ant attendance treatment), we selected 54 trees containing nests of *C. macaoensis*. In these trees, numerous workers of *C. macaoensis* constantly attended *K. yunnanensis* both day and night, excluded all other ant species, and often constructed protective shelters over *K. yunnanensis* [22], [30].

If our two treatments merely varied the abundance of a single ant species we would anticipate having a single relationship between parasitoids and the ants of the two ant attendance treatments. Instead, because there are different ant species within the two ant attendance treatments, we would anticipate multiple relationships. This more complicated design reflects the on-ground reality of the farming system and therefore has greater practical application.

As far as practicable, we attempted to create and utilize equivalently sized populations (hereafter called aggregations) of *K. yunnanensis*, but this was not necessarily the case and could not be confirmed for multiple reasons. First, although brood lac were of approximately equivalent size, they did not necessarily contain the same number of *K. yunnanensis* larvae. Second, the larvae are initially mobile and can move among aggregations, so the number of insects within aggregations can vary greatly. Third, because *K. yunnanensis* are covered by lac, it is not possible to directly count the number of individuals in the aggregations. However, in the absence of predators and parasitoids *K. yunnanensis* is known to have the same population density per cm^2^ of lac scale on the same host tree species [31]. Consequently, after parasitoid collection we measured the area of the lac crusts to confirm uniformity of sampling.

### Parasitoid sampling

Parasitoids emerging from lac crusts were trapped in February and April 2010 using 25 cm long×10 cm diameter polyester mesh (1 mm^2^) cylinder traps placed around individual crusts (Figure S1). These timings corresponded with the final *K. yunnanensis* larval stage, and middle-end adult stage respectively, and were chosen because it was anticipated that the parasitoid composition would differ at these two *K. yunnanensis* developmental stages [31]. Crusts were selected on the basis that (1) they were longer than 10 cm, but in a few instances some crusts were slightly smaller, (2) there were no external signs of the presence of *K. yunnanensis* predators, and (3) the ant abundance on the crusts, prior to trap placement, conformed to the designated ant attendance treatment of the tree.

Ant abundance counts were performed prior to setting the parasitoid traps by counting the number of ants displaying tending behavior (e.g. protection, feeding, not just walking across the crust) over a 10 second period. Prior to placing a trap around each crust, the crust surface was cleared of all insects using a sharp blow of breath. Traps were operated for one month to ensure all parasitoids in each crust were captured. Only one trap was used per tree, except for six samples from the high-ant attendance treatment where two crusts on different branches were sampled in different sample times to obtain sufficient replication. In all, we collected 30 replicates per treatment per sample time. All adult parasitoids were counted and identified to the species level using keys in [32].

### Analyses

Except when stated otherwise, analyses were conducted using STATISTICA 11. Not all *K. yunnanensis* aggregations were parasitized and there was no pattern of lack of parasitism with treatment (e.g. all non-parasitized aggregations being in the high ant attendance treatment, or increasing lack of parasitism with decreasing ant attendance), so we excluded such trees from statistical analyses, leaving 22, 20 and 21 samples (February) and 28, 29 and 22 samples (April) in the exclusion to high ant attendance treatments respectively.

**Figure 1:**
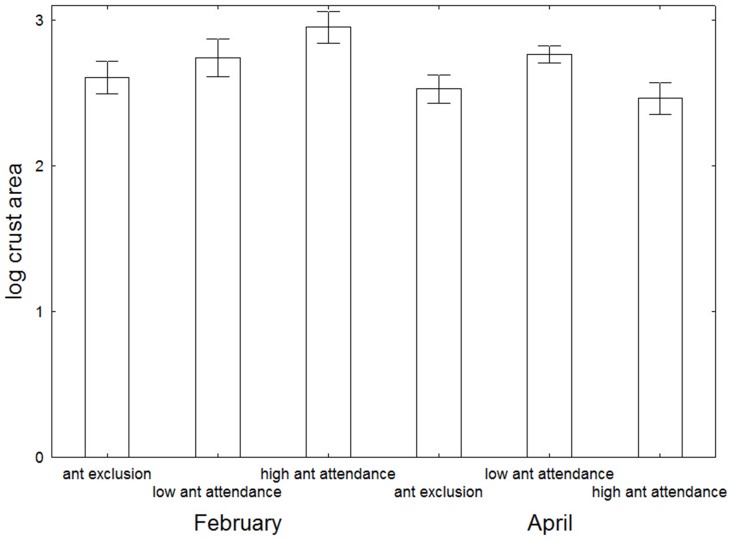
Mean (±SE) lac crust area sampled in the three treatments and two sample times. Data are log transformed.

To confirm uniformity of sampling, the area of lac crust sampled for parasitoids among treatments and sample times was compared using a Two-way ANOVA of log transformed data. Although these data did not quite satisfy Levene's test (P = 0.039), visual inspection of the residuals found no issue, so we used this method because of its far greater sensitivity compared to non-parametric tests. Overall there was no difference among treatments (2-way ANOVA; F = 1.9, P = 0.15), but there was a difference for time (F = 4.7, p = 0.03) and the interaction (F = 3.4, P = 0.04). This was because the unusually hot summer resulted in the death of many *K. yunnanensis* aggregations, especially in the high ant attendance treatment, so samples in April across treatments did not follow the same size pattern collected in February (Figure 1).

To compensate for the slight non-homogeneity of sample size, we initially standardized the ant and parasitoid data per crust area, however, we found no relationship between ant abundance or parasitoid abundance with crust area. Instead it appears that ants merely respond to the presence/absence of crust, and the effects on parasitoids are dependent on the type and number of ants present, irrespective of the size of the crust. Additionally, the patterns of parasitoid abundance per crust and their statistical separation were identical when using non-standardized and standardized data. Accordingly we used unadjusted abundance data for both ants and parasitoids in all analyses, and acknowledge that sample surface area of crusts between sample times are not fully homogeneous.

Non-metric multidimensional scaling was used to compare homogeneity of ant community structure of the low ant attendance treatment between the two sample times, and parasitoid community structure among the three treatments in the two sample times. The association matrices were based on a Bray-Curtis association of species-level abundance data per crust of ants and parasitoids respectively. Differences among categories were tested using Analysis of Similarity (ANOSIM) for the ant data, and PERMANOVA for the parasitoid data. All multivariate analyses were conducted using Primer 6.

Because parasitoid abundance and species varied so greatly between the two sample times, different statistical tests were used for different levels of analysis. Total parasitoid abundance data were compared among treatments and between sample times using 2-way ANOVA, and Tukey's HSD test for the post-hoc comparison of means. The abundance data was log (x+1) transformed so that residuals met parametric test assumptions, with normality and homogeneity of variance being assessed using graphical observations and Levene's test respectively.

Abundance data of individual species could only be assessed for the three most common species, but not always for both sample times. Additionally these data could not satisfy assumptions for parametric tests. For consistency of tests, where there were enough data to perform a test, we analysed sample times separately using non-parametric Kruskal-Wallis ANOVA.

Parasitoid species richness per crust varied little and was heavily skewed, so we compared parasitoid species richness among treatments and between sample times using a poisson-log generalized linear model.

For analyses where ant abundance was used as a continuous variable, we excluded the data from the ant exclusion treatments, because in all but one case the abundance of each parasitoid species in the absence of ants covered more than the full extent of abundance found with the presence of ants, indicating that there are different drivers determining parasitoid abundance with and without ants resulting in different relationships. Additionally we consider the data for the low and high ant attendance treatment separately because they have clearly different relationships indicating that the different ant species in the two treatments affect parasitoids differently. Finally, we merged the data of the two sample times for the high ant attendance treatment because only a single ant species is within this treatment and the data fall clearly under one relationship. However we consider the two sample times separately for the low ant attendance treatment because the ant species present in the two times were varied greatly, and produced different relationships with parasitoid abundance. Power and exponential regressions were used to determine the relationship between the number of attending ants and total parasitoid abundance, as well as with the abundance of the three most abundant parasitoids. The best fit was determined using Akaike's Information Criterion (AIC), which favors both model fit and model simplicity. Regressions and AICs were conducted using SPSS v16.

## Results

**Figure 2:**
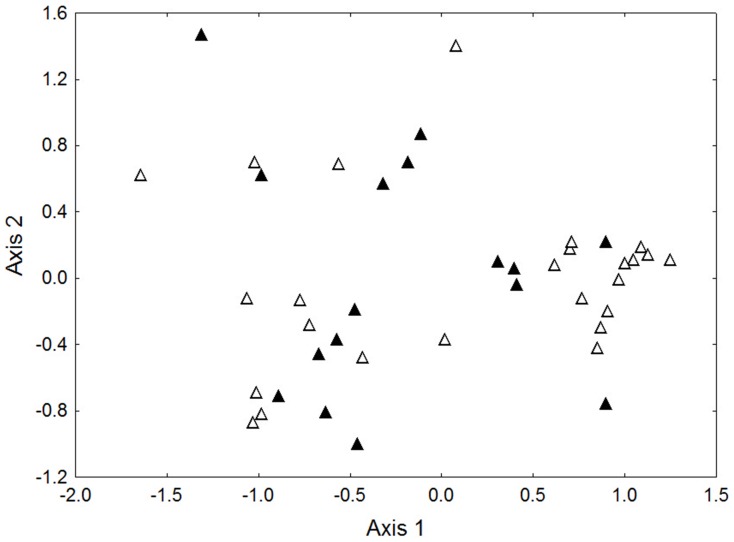
NMDS ordination of species-level ant abundance data. Data are for low attendance treatment only, sampled in February (closed symbols) and April (open symbols). 2D stress  = 0.1

Within the low ant attendance treatment we found 11 species tending crusts, six in February, and eight in April (Table S1). The two most abundant species were *Crematogaster ferrari and C. macaoensis*, contributing 50.8 and 30.5% of individuals respectively. Ordination of the species-level ant abundance data showed that the species and their abundances differed significantly between the two sample times (Figure 2; ANOSIM Global R = 0.133, P = 0.005). This is largely due to the February sample having approximately half (5) the number of ants per crust compared to the April sample (10), and the greater presence of the two most common ants, *C. ferrari* and *C. macaoensis* in April compared to February (present on 86% of crusts vs 65% respectively).

**Figure 3:**
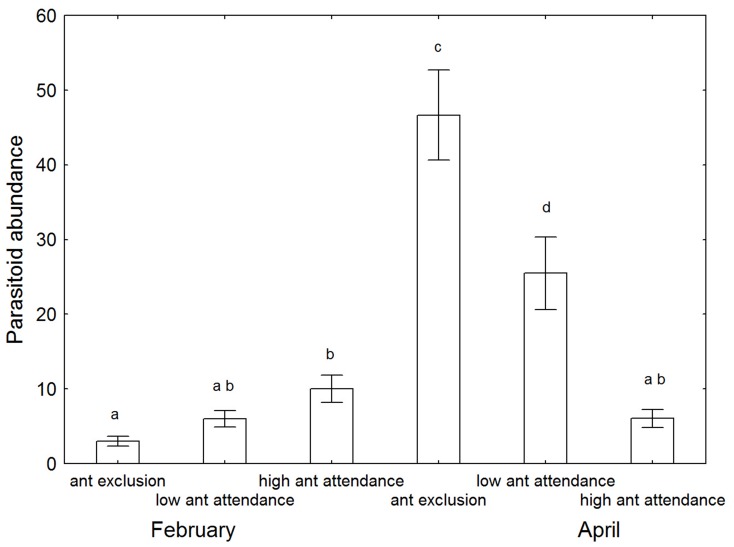
Mean (±SE) parasitoid abundance in the three treatments and two sample times. Letter codes indicate statistical separation.

A total of 2574 individuals of parasitoids belonging to five species were collected (Table 1). The two most abundant species were *Tetrastichus purpureus* and *Tachardiaephagus tachardiae*, contributing 82.7% and 13.2% of individuals respectively. Total parasitoid abundance varied greatly with treatment (Two-way ANOVA; F = 4.4, P = 0.014) and sample time (F = 52, P<0.0001), with the two factors having a strong interaction effect (F = 33, P<0.0001). Total parasitoid abundance was lowest in the February sample when *K. yunnanensis* was in its younger life stage, and interestingly was significantly lower in the ant exclusion treatment (Figure 3). In April, all three treatments had significantly different parasitoid abundances (Figure 3), with abundance being highest in the ant exclusion treatment and the lowest in the high ant attendance treatment.

**Table 1:** Mean (±SE) parasitoid species abundance data within the three ant attendance treatments from the two sample times.

Family	Species	Treatment					
		sample	Ant exclusion	Low ant attendance	High ant attendance	H	P
Eulophidae	*Tetrastichus purpureus*	February	3.6±1.2^a^	3.6±0.8^a^	1.3±0.3^a^	2.89	0.278
		April	44.1±5.9^a^	24.7±4.8^b^	4.8±1^c^	33.03	<0.0001
	*Marietta javensis*	February	0	0	0		
		April	7±4.2^a^	2.6±0.5^a^	8±0^a^	1.88	0.39
Aphelinidae	*Coccophagus tschirchii*	February	0	3±0	1±0		
		April	0	0	0		
Encyrtidae	*Tachardiaephagus tachardiae*	February	1.8±0.4^a^	4.7±1.3^ab^	9.1±1.9^b^	12.25	0.002
		April	2.4±0.6^a^	1.5±0.2^a^	2.7±0.8^a^	2.59	0.274
Eupelmidae	*Eupelmus tachardiae*	February	0	0	2.4±0.6		
		April	0	0	0		

Letters indicate statistical separation among treatments within sample time as found by Kruskal-Wallis ANOVAs. Blank areas indicate no statistical test was conducted.

**Figure 4:**
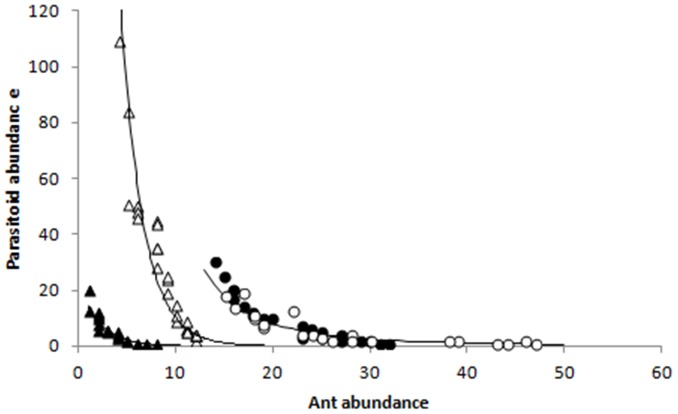
Relationship between number of tending ants and total parasitoid abundance. Treatments and sample times are low (triangles) and high (circles) ant attendance treatments sampled in February (closed symbols) and April (open symbols). Relationship metrics are: low ant attendance February sample: y = 22.627e^−0.459x^, R^2^ = 0.9257; low ant attendance April sample: y = 831.1e^−0.439x^, R^2^ = 0.914; high ant attendance: y = 39371x^−2.837^, R^2^ = 0.8571.

**Figure 5:**
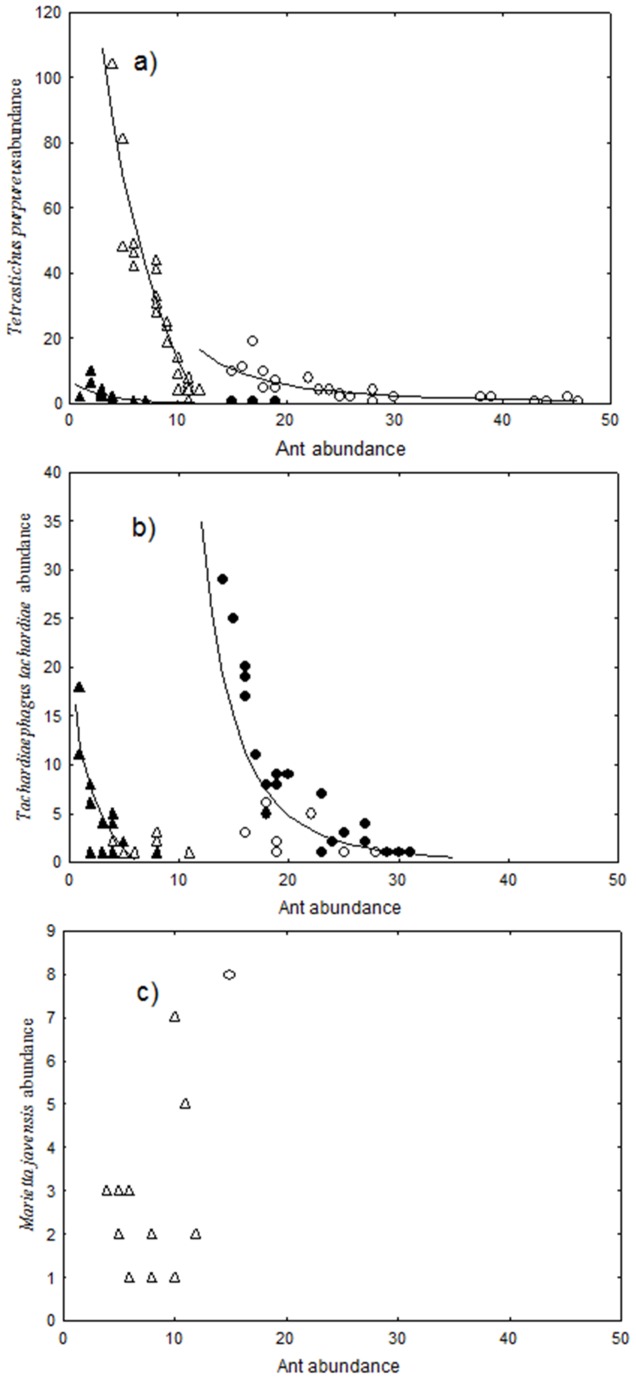
Relationship between number of tending ants and the three most abundant parasitoid species. Treatments and sample times are low (triangles) and high (circles) ant attendance treatments sampled in February (closed symbols) and April (open symbols). Relationship metrics are: graph a) low ant attendance February sample: y = 70277e^−0.301x^, R^2^ = 0.464; low ant attendance April sample: y = 839.3e^−0.454x^, R^2^ = 0.827; high ant attendance: y = 2930.6x^−2.079^, R^2^ = 0.753; graph b) low ant attendance February sample: y = 10.316x^−1.128^, R^2^ = 0.496; high ant attendance: y = 523271x^−3.87^, R^2^ = 0.638.

When ants were present, there were strong negative relationships between total parasitoid abundance and ant abundance, with the relationships being dependent upon the ant species composition and abundance (Figure 4). For the single species *C. macaoensis*, its relationship with parasitoid abundance was consistent between the two sample times. However, the ant assemblages of the low ant attendance treatment that differed between the two sample times (Figure 2) displayed distinctly different relationships with parasitoid abundance (Figure 4). The patterns of total parasitoid abundance were clearly driven by the two most abundant parasitoid species (Figure 5a,b). No relationship was evident for *Marietta javensis*, the only other species with enough data to analyse (Figure 5c).

**Figure 6:**
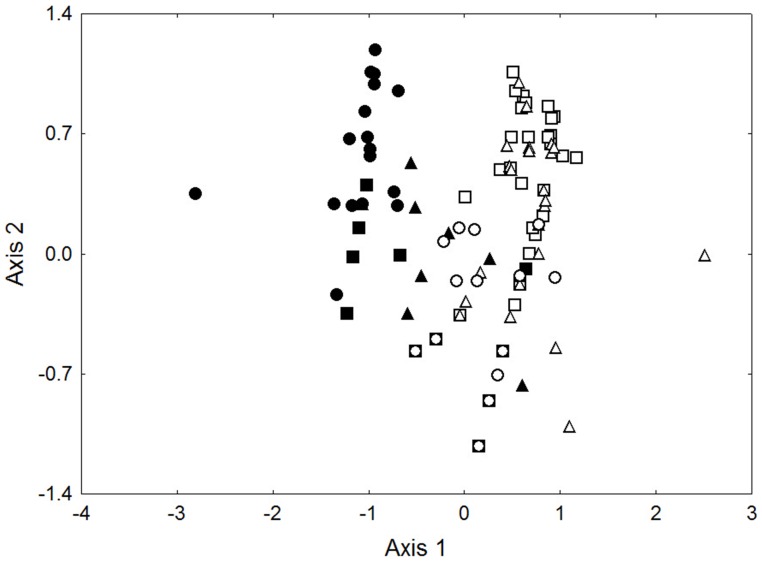
NMDS ordination of species-level parasitoid abundance. Treatments and sample times are low (triangles) and high (circles) ant attendance, and ant exclusion (squares), sampled in February (closed symbols) and April (open symbols). 2D stress  = 0.1.

Parasitoid species richness did not differ among treatments (GLM; X^^2^^ = 0.79, P = 0.67), or between sample times (X^^2^^ = 0.69, P = 0.41). However, ordination of species-level parasitoid abundance data confirmed that overall parasitoid community structure differed significantly among treatments and between sample times (Figure 6; PERMANOVA: P<0.0001 for all categories and interactions), with pairwise tests finding all comparisons differing significantly (P<0.019), with the high ant attendance treatment differing most from the other two treatments (P<0.0001). These community-level differences were a result of great differences at the species-level (Table 1). *Tachardiaephagus tachardiae* was most common in February, and was largely replaced in April by *T. purpureus*. *Marietta javensis* was only present in April, predominantly only in the ant exclusion and low ant attendance treatments. The other two species were caught in a single sampling period, and with too few individuals to identify patterns, but interestingly were always found in treatments with ants.

## Discussion

Our results clearly showed strong negative relationships between the abundance of tending ants and parasitoids, with the relationships being dependent upon the ant species present, ant abundance, and the *K. yunnanensis* developmental stage. Greatest parasitism rates occurred at the oldest developmental stage measured, with the rate being least where *Crematogaster macoensis* were present in high abundance.

Asymmetric protection from enemies provided to symbionts by different ant species is well documented globally [7], [10], [33], [34] with greater protection being provided by ants with greater abundance and greater aggression. Here, *C. macaoensis* was the most abundant, and the most aggressive species, and it provided the greatest protection from parasitoids possibly because they build structures over the lac crusts that provide additional protection against parasitoids. So from the perspective of both *K. yunnanensis*, and the farmer, *C. macaoensis* is best to protect lac insects within this agricultural system.

Interestingly *C. macaoensis* belongs to the subfamily Myrmicinae, which is counter to the global trend of the most common species of ant tending honeydew-producing insects being members of the subfamilies Formicinae and Dolichoderinae. This global phenomenon is probably largely due to the morphological adaptations required in ant gut systems to allow ants to carry large amounts of liquid honeydew, and Myrmicinae are among the ants with the least modified guts [35]. However, Myrmicinae are capable of attaining high abundance levels, and have chemical defenses, and so are capable of dominating resources in the absence of high-tempo and aggressive species from other ant subfamilies [36], [37].

Another interesting observation was that the two uncommon parasitoid species were never found within crusts from the ant exclusion treatment, and for only one species in one sample time, *T. purpureus* in April, was its abundance significantly greater within crusts from the ant exclusion treatment. Thus the absence of ants did not result in increased parasitism for four of the five parasitoid species. Two, potentially interrelated explanations are possible. First, parasitoids find *K. yunnanensis* hosts indirectly through the presence of ants, rather than directly. This would also explain why the second most abundant parasitoid species, *T. tachardiae*, was most abundant in the two ant attended treatments in the February sample when *T. purpureus* abundance was low. Such host detection has not been postulated previously and warrants further attention. Second, the most abundant parasitoid, *T. purpureus*, is able to competitively exclude other parasitoids from high-quality, unprotected crusts. There was a clear difference in the levels of parasitism between the February and April samples, so there was also likely to have been a big difference of competitive pressure among parasitoids. In February, when *K. yunnanensis* were in their youngest growth stage, parasitism rates were low, and so it is likely that there was little competitive pressure among parasitoids during this period. However, by April parasitism rates were much greater, and thus competition for access to unprotected crusts must have also been great, and the abundance of *T. purpureus* relative to all other parasitoids suggests that it is capable of outcompeting these species.

In addition to ant-mediated parasitism rates, implicit in the experimental design is that differences in parasitoid community composition among treatments is also due to the composition and abundance of tending ants. Parasitoid communities being influenced by ant species is a known phenomenon [38], and would be expected in every instance where multiple parasitoid species live in sympatry, due to asymmetrical interaction outcomes between different pairs of ant and parasitoid species. Greater insight, and ultimately a predictive understanding of the complexities of ant-parasitoid-host dynamics will require further experimental manipulation to assess the relative roles of 1) the abundance of each individual ant species on parasitoid access to hosts, 2) competition among parasitoids, and 3) the interaction between the first two factors.

More broadly for theoretical ecology, several hypotheses have been offered to explain mutually beneficial interactions between ants and honeydew-producing insects. Our results supported the predictable rewards hypothesis [39], [40]. For ants, the honeydew secreted by *K. yunnanensis* was predictable in space and time, which elicited repeated visits by ant foragers, and incidentally increased the likelihood of encounters with parasitoids without requiring an increase in ant activity or aggressiveness.

Clearly ant-lac insect-parasitoid relationships in the agroecosystems of SE China are complex. Additional manipulative research, especially focused on fine-scaled relationships, is needed to provide a predictive understanding of the interplay of species in this dynamic system. Such work will undoubtedly increase the recognition of the beneficial role of ants within agriculture, and improve the use of ants as an environmentally friendly crop protection strategy [41], [42], [43], [44].

## Supporting Information

Figure S1 Parasitoid trap, consisting of a 25 cm long×10 cm diameter polyester mesh (1 mm^2^), was placed on the lac crust to collect parasitoids.
(PDF)

Table S1 Parasitoid species and abundance captured from certain area of lac crust within the three ant attendance treatments from the two sample times, which were used in the analyses presented in the paper.
(XLSX)
