# Supplementary material for: Multiple Ant Species Tending Lac Insect Kerria yunnanensis (Hemiptera: Kerriidae) Provide Asymmetric Protection against Parasitoids
Source: PLoS One. 2014 Jun 2;9(6):e98975. doi: 10.1371/journal.pone.0098975 (PMC4041774; doi:10.1371/journal.pone.0098975)

1. A lac crust.

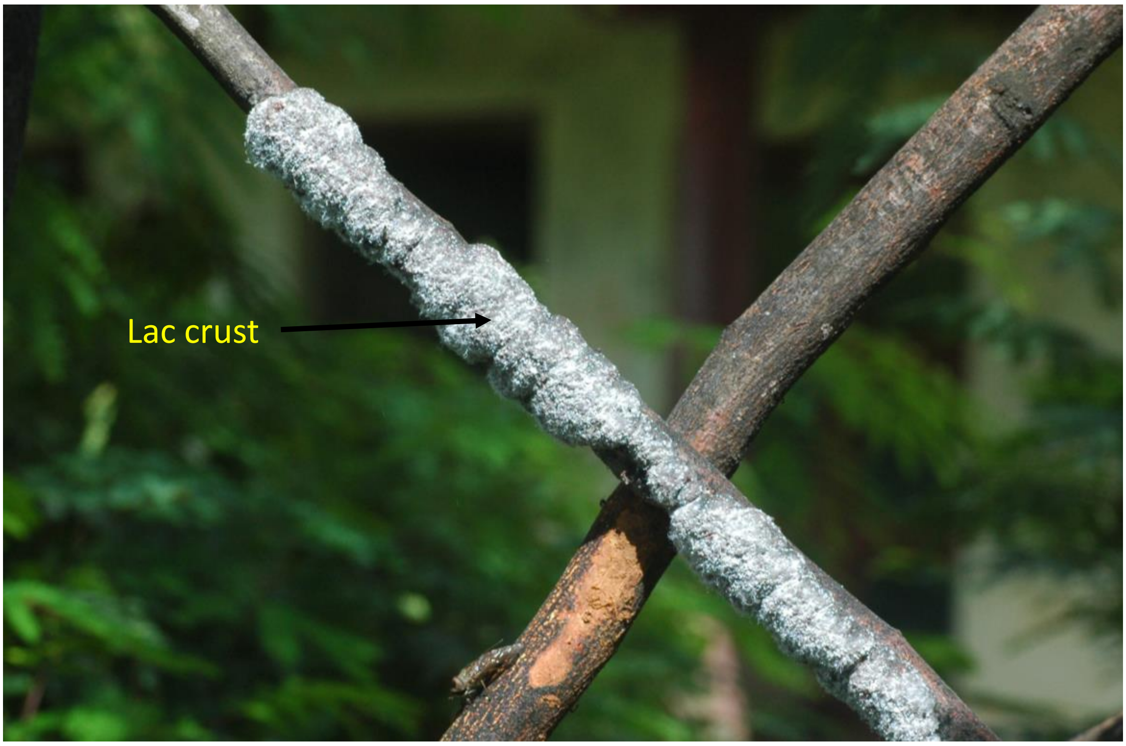

2. Parasitoid trap, consisting of a 25 cm long x 10 cm diameter polyester mesh (1mm^2)

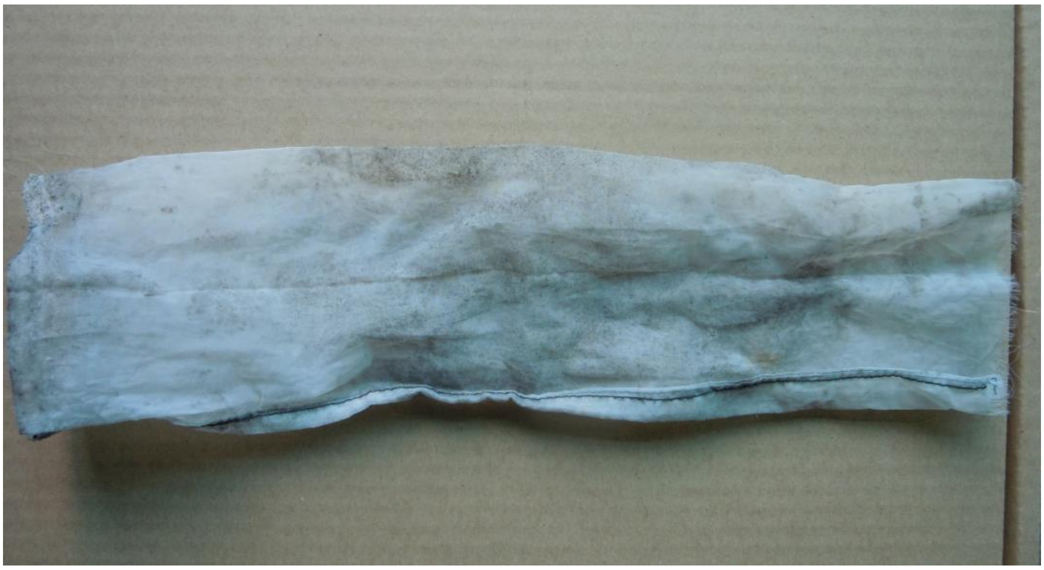

3. Traps placed on the lac crusts on the tree.

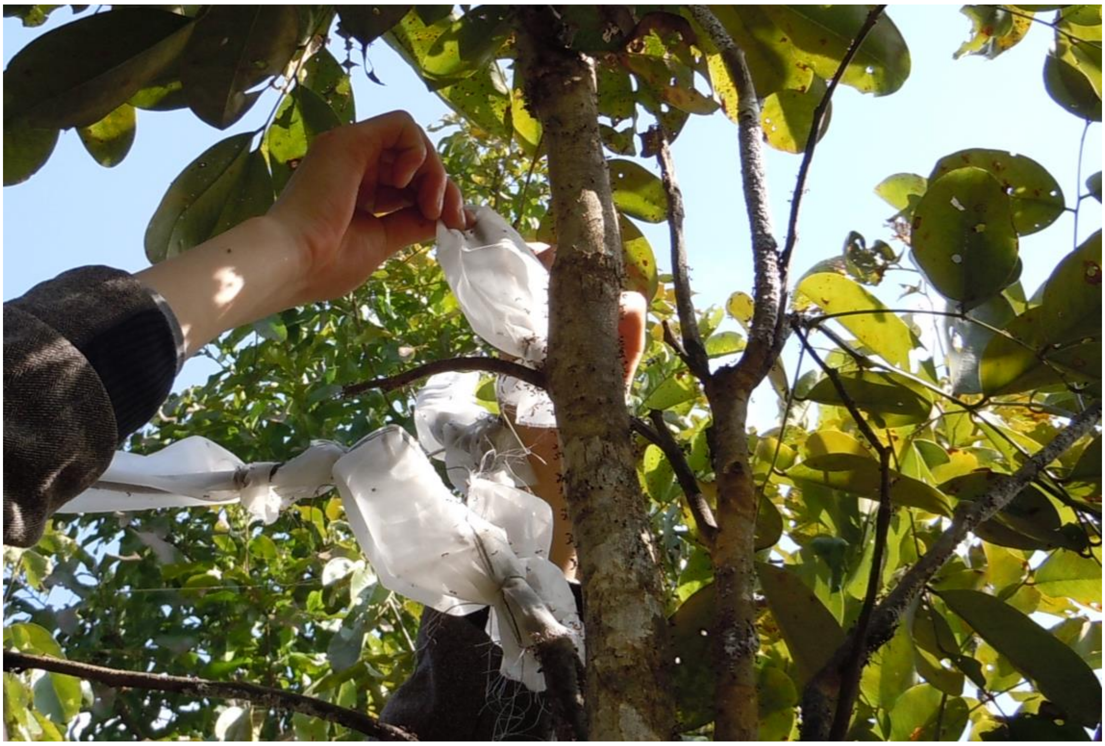

Supplement: Figure S1 — Parasitoid trap, consisting of a 25 cm long×10 cm diameter polyester mesh (1 mm2), was placed on the lac crust to collect parasitoids. (PDF) [file pone.0098975.s001.pdf]
